# Simple Method for Establishing Primary Leporidae Skin Fibroblast Cultures

**DOI:** 10.3390/cells10082100

**Published:** 2021-08-16

**Authors:** Fábio A. Abade dos Santos, C. L. Carvalho, Isabel Almeida, Teresa Fagulha, Fernanda Rammos, Sílvia C. Barros, Margarida Henriques, Tiago Luís, Margarida D. Duarte

**Affiliations:** 1 Centro de Investigação Interdisciplinar em Sanidade Animal (CIISA), Faculdade de Medicina Veterinária, Universidade de Lisboa, Avenida da Universidade Técnica, 1300-477 Lisboa, Portugal; margarida.duarte@iniav.pt; 2 Instituto Nacional de Investigação Agrária e Veterinária (INIAV, I.P.), Av. da República, Quinta do Marquês, 2780-157 Oeiras, Portugal; carina.carvalho@iniav.pt (C.L.C.); isabel.almeida@iniav.pt (I.A.); teresa.fagulha@iniav.pt (T.F.); fernanda.ramos@iniav.pt (F.R.); Silvia.SantosBarros@iniav.pt (S.C.B.); margarida.henriques@iniav.pt (M.H.); tiago.luis@iniav.pt (T.L.); 3 Instituto Universitario de Biotecnología de Asturias (IUBA), Departamento de Bioquímica y Biología Molecular, Universidad de Oviedo, 33003 Oviedo, Spain

**Keywords:** primary fibroblasts, primary cell culture, virus isolation, method, dispase II, Leporidae

## Abstract

Commercial hare and rabbit immortalized cell lines are extremely limited regarding the many species within the lagomorpha order. To overcome this limitation, researchers and technicians must establish primary cell cultures derived from biopsies or embryos. Among all cell types, fibroblasts are plastic and resilient cells, highly convenient for clinical and fundamental research but also for diagnosis, particularly for viral isolation. Here, we describe a fast and cheap method to produce primary fibroblast cell cultures from leporid species, using dispase II, a protease that allows dermal–epidermal separation, followed by a simple enzymatic digestion with trypsin. This method allows for the establishment of an in vitro cell culture system with an excellent viability yield and purity level higher than 85% and enables the maintenance and even immortalization of leporid fibroblastic cells derived from tissues already differentiated.

## 1. Introduction

Leporids comprise different species of hares and rabbits that have drawn the attention of the scientific community (e.g., as human diseases models, to produce immune sera and monoclonal antibodies, for example), the farming sector, the general public, and conservationists for very different reasons. These range, from its use as models for human diseases, livestock for human consumption, pets, game species, and for their notorious role in the preservation of important ecosystems and biodiversity. Since leporid pathogens can impact a wide range of areas and sectors, the study of their diseases is of paramount importance. Viral isolation is a basic tool in the diagnosis of well-described and unknown viruses, as well as for fundamental research that enables the understanding of the pathogenesis of several diseases through the study of every step that defines the virus–host cell interactions. Moreover, these interactions require in vitro cultured cells permissive to the infection, which allows the binding of viral capsid (for naked viruses) or viral envelope glycoproteins to the receptors present at the cell membrane, which are specific to each cell phenotype.

Primary cells are obtained regularly from living tissues, most often from embryonic sources, and are subsequently submitted to in vitro culture procedures. Despite some disadvantages when compared to immortalized cell lines, namely the inability to survive after a limited number of in vitro passages [1], primary cells preserve the same characteristics as those from the originating tissue, facilitating studies in many research areas, namely in virology and cell biology that otherwise would be far more complicated. Additionally, they are relatively cheap and easy to obtain and may be prepared from any animal species.

The hare and rabbit immortalized cell lines currently available are limited to epithelial-like kidney cells (RK13 from rabbit and HN-R and a few more from European hare), and to skin primary fibroblasts from New Zealand rabbits that are also commercially available. However, the specificity of certain studies requires the preparation of primary cell cultures from the host under analysis. This is the case, for instance, for skin fibroblasts useful for investigating Iberian hare (*Lepus granatensis*) viruses.

Fibroblasts are mesenchymal-derived cell types, involved in the synthesis of extracellular matrixes (ECMs), such as collagen, elastin, fibronectin, and others, and the secretion of growth factors. They play a central role in maintaining structural homeostasis as water-holding glycoproteins [2].

Fibroblasts are frequently used to study cellular biological processes, for clinical research, toxicological studies, microbiological investigations, among others. In the virology field, cultured fibroblasts are very useful for primary isolation and the adaptation of wild type virus strains since the number of viral particles in infected tissue samples obtained from primary or secondary replication organs in field isolates is usually low. Furthermore, viruses do not grow in cells derived from heterologous species, even when expressing the appropriate receptor [3,4]. Fibroblasts cell cultures are one of the easiest to be established, as no complex purification processes are required and, depending on the species of origin, cells can even be prepared from samples collected from live animals without need of anesthesia, sedation, or sacrifice. This is the case for Leporidae. There are many protocols available with different levels of complexity, approaches, and objectives, some of which are summarized in Table 1. The protocol presented here is the first optimized for obtaining primary fibroblasts from live wild leporids. The method is simple, low cost and enables large amounts of cells to be obtained and, therefore, worthy of sharing. The protocol uses dispase II, a proteolytic enzyme, followed by a simple incubation with trypsine solution. Despite the method having been implemented and optimized for Iberian hare (*Lepus granatensis*) and wild rabbit (*Oryctolagus cuniculus algirus*) fibroblast cultures, it can be applied to biological samples of any species, proving to be easy and quick to perform.

## 2. Material and Methods

### 2.1. Tissue Sample Collection

**Figure 1:**
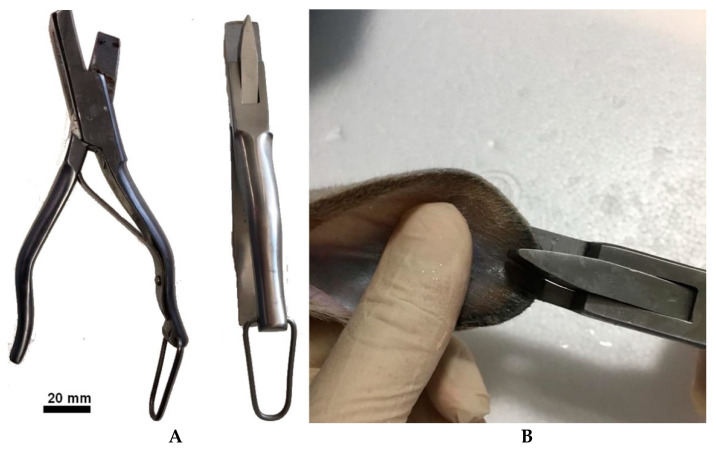
(**A**) Commercial equipment used for skin sample. (**B**) Collection of an ear sample.

All biological samples used in this study were collected from wild Leporidae in captive centers by skin punch biopsy for genetic analysis or during animal marking. The tool used is commercially available (Figure 1A). This study was conducted in the scope of a project called +Coelho that operationalizes the Action Plan for the Control of Rabbit Hemorrhagic Viral disease in Rabbits (Dispatch 4757/17 of 31 May), specifically approved by the Portuguese National Authority for Animal Health (Authorization 79/ECVPT/20145). No animals were subjected to housing or handling for the exclusive purpose of this study. All sampling procedures were performed by veterinarians.

After collection in the field or in the animal premises, the tissue samples were stored in culture medium (DMEM/HAMS F12 medium with L-Glutamine and HEPES (3.5 g/L) (CORNING), supplemented with 200 units/mL of penicillin, 200 µg/mL of streptomycin, 0.5 µg/mL of Gibco Amphotericin B, and 50 µg/mL of gentamicin) at room temperature (RT) for a maximum of two hours, and then incubated at 37 °C for a maximum of three hours prior to preparation. For longer storages, 50-milliliter Falcon tubes or T25 culture flasks are recommended. During storage at 37 °C, the medium should be replaced every hour.

Ideally, skin samples should be obtained from healthy juvenile animals, rather than adults. However, this protocol was successfully used with adult skin samples.

Before harvesting, asepsis of the biopsy ear area was carried out by seven washes with a chlorhexidine solution using swabs, followed by one wash with sterile 0.9% NaCl. The biopsy of the ear was performed using a biopsy punch (Figure 1B). Samples were taken from the margin of the ear, avoiding major blood vessels that can easily be visualized against light. If correctly performed, no hemorrhage, pain, inflammation, or infection is produced during and after biopsy.

In docile animals (e.g., domestic rabbits) the area can be clipped, but this procedure should be avoided in wild animals sensitive to clipping noise and vibration.

### 2.2. Reagents

For this protocol only two reagents are required, namely dispase II (42613-33-2, Sigma-Aldrich, St. Louis, MO, USA) and a trypsin dissociation solution (0.8% NaCl, 5 mM KCl, 5.5 mM Glucose-Dextrose, 7 mM Na_2_HPO_4_, 1.25 g of Trypsin 1:250, Sigma-Aldrich, pH 7.2 ± 0.2, filtered using a 0.22-micrometer mesh).

### 2.3. Preparation of Fibroblasts from Skin Biopsy

All steps should be performed in a laminar flow hood using sterile using sterile disposable material.

**Figure 2:**
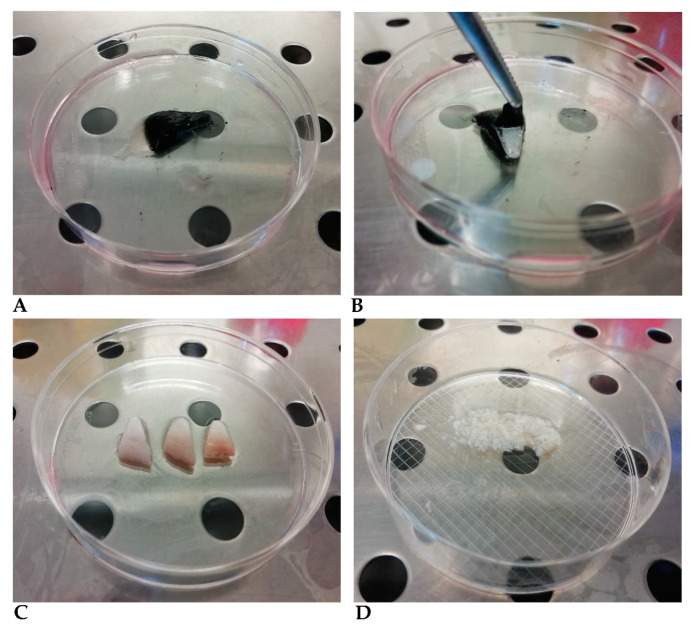
(**A**) Ear sample before dispase II treatment, (**B**) Ear sample after incubation with dispase II, showing the detach of the epidermis, (**C**) Ear sample after epidermis removal, (**D**) Dermis fragments cut to less than 5 mm diameter dimensions.

(1) Wash the original tissue fragments to remove impurities by placing the sample in a 50-milliliter Falcon tube containing 30 mL DMEM/HAMS F12 medium with L-Glutamine and HEPES (3.5 g/L) (CORNING), 5× Antibiotic–Antimicotic solution (200 units/mL of penicillin, 200 µg/mL of streptomycin, 0.5 µg/mL of Gibco amphotericin B and 50 µg/mL of gentamicin); immediately proceed with vigorous washing, using a shaking robot or just manual shaking. Change medium seven times after 1-min-long agitation.
(2) Place the original tissue fragments in 50-milliliter Falcon tubes (or alternatively in T25 bottles, or Petri dishes) and fill with DMEM medium prepared as described below.
(3) Remove the medium and add a volume of 0.2 mg/mL dispase II prepared in DMEM medium enough to cover the tissue fragments. The epidermis–dermis separation process takes a variable time depending on the dimension, shape, and thickness of the fragments, generally 1–2 h at 37 °C. We recommend shortening this procedure as much as possible, since hair and epidermis are contaminated by bacteria, fungi, and yeasts, even after washing, and must be removed from the preparation as soon as possible. The operator must, therefore, test the detachment every hour, although cells may be left overnight in this medium with no adverse effects. Remove the epidermis completely using two tweezers (Figure 2B,C) and wash the epidermis fragment with DMEM medium as often as necessary until no hairs are visible (Figure 2C).
(4) Cut the original fragments into smaller fragments less than 5 mm wide, using a scalpel (Figure 2D).
(5) Incubate the fragments with the trypsin dissociation solution (the smallest volume that covers the fragments), previously warmed to 37 °C, in 50-milliliter tubes, T25 culture flasks or Petri dishes, and incubate at 37 °C for 10 min. The dissociation solution becomes cloudy as cells detach. Caution must be taken at this step since trypsin activity continues beyond 30 min, destroying the cells.
(6) Remove the enzymatic digestion solution containing the cells and centrifuge at 150× *g* for 10 min. Resuspend the cells in a conical tube containing DMEM/HAMS F12 medium with L-Glutamine AND HEPES (3.5 g/L), 5× antibiotic-antimycotic solution (100 units/mL of penicillin, 100 µg/mL of streptomycin, 0.5 µg/mL of Gibco amphotericin B, and 25 µg/mL of gentamicin) and 10% of fetal bovine serum (Sigma-Aldrich, St. Louis, MO, USA).
(7) Add fresh dissociation solution to the tissue fragments and repeat steps (5) and (6) as many times as necessary.
(8) Seed the cells in culture flasks. Incubate at 37 °C with 5% CO_2_.
(9) Remove medium after 2–4 h incubation, wash the cells with DMEM, and add fresh medium.
(10) Remove medium again after 2–4 h of incubation. Wash the cell layer with DMEM and add fresh medium.

The presence of dead cells in suspension during the first 48 h of incubation is expected. Due to the normal skin microbiome, some cultures may show signs of contamination after 24–48 h incubation and, therefore, must be eliminated. In plastic surfaces where the initial cell density is low, supplementation of the culture media with 20% fetal bovine serum is recommended in order to assure cell viability and turnover of mitosis. The percentage of fibroblasts in culture using this technique exceeds 85% (evaluated using morphological analysis).

**Figure 3:**
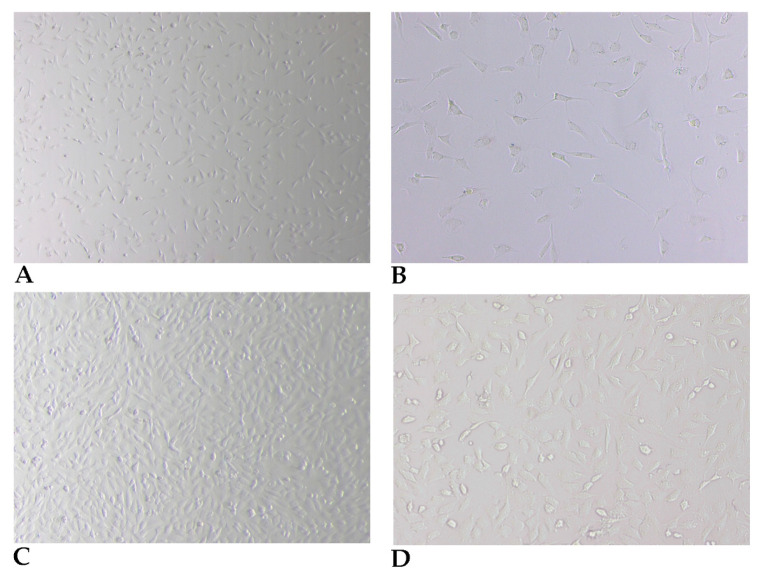
Phase contrast microscopy of typical fibroblast cells after step (7) of the *Preparation of fibroblasts from skin biopsy* procedure, at day 2, 40× (**A**) and 100× (**B**), and at day 4, 40× (**C**) and 100× (**D**).

Plated cells (Figure 3A,B) assume the typical spindle-shaped morphology and reach 60 to 70% confluence between days 2 and 3 of incubation. Although the purity of these cultures in fibroblasts reaches proportions of 75% or higher, morphologically distinct cells, namely epithelioid or stellate-like cells, are observed in the cultures using optical-phase microscopy. The different morphologies assumed by the fibroblasts are shown in Figure 3C,D.

### 2.4. Subculturing and Harvesting Primary Fibroblast Cells

Fibroblast cells have a high multiplication ratio and must be subcultured when the cell layer reaches about 90% confluence.

(1) Remove the medium and wash cells twice with sterile PBS.
(2) Incubate cells with trypsin-EDTA (0.25%), enough to cover the cell layer, and incubate at 37 °C until cell detachment.
(3) Centrifuge the cell suspension at 150× *g* for 10 min and recover the pellet.
(4) Cells are counted in a Neubauer hematocytometer and plated at 1–2 × 10^4^ cells/cm^2^

Note: Fibroblasts are not contact inhibited. The primary fibroblasts are particularly sensible to the trypsinization (comparing with typical immortalized cell lines); therefore, their viability is generally lower.

### 2.5. Viability Assay Using Trypan Blue Dye

Viability of cells after cryopreservation was evaluated by cell counting after trypan blue staining and when necessary, after subculturing and freezing process. Cells were diluted at 1:10 in Trypan Blue, counted in a Neubauer chamber, and frozen.

### 2.6. Freezing and Thawing Fibroblast Cells

Cryogenic preservation can maintain fibroblast cells for very long periods [9]. Subcultures with 3 or 4 passages should be used for cryogenic preservation.

(1) Trypsinise cells with 70–80% confluence, as described above in Section 2.4 (steps (1)–(3)).
(2) Centrifuge at 150× *g* for 10 min and resuspend the cell pellet with 10% DMEM, 10% DMSO, and 80% FBS (*v*/*v*).
(3) Aliquot the cell suspension in cryogenic storage tubes at a density of 1–3 × 10^6^ cells/vial. We have obtained an average of two vials for every three T25 flasks with 90% confluence.
(4) Freeze cells at −80 °C overnight and then transfer them into liquid nitrogen.

To resuscitate cells after liquid nitrogen freezing, vials must be quickly thawed at 37 °C and promptly transferred to cell culture flasks with DMEM medium. After 12–24 h of incubation, wash cells, and add fresh medium.

If strict asepsis techniques are used, it is possible, and recommended, to maintain these cultures without the addition of antibiotics.

### 2.7. Fibroblast Marker by Immunocytochemistry

**Figure 4:**
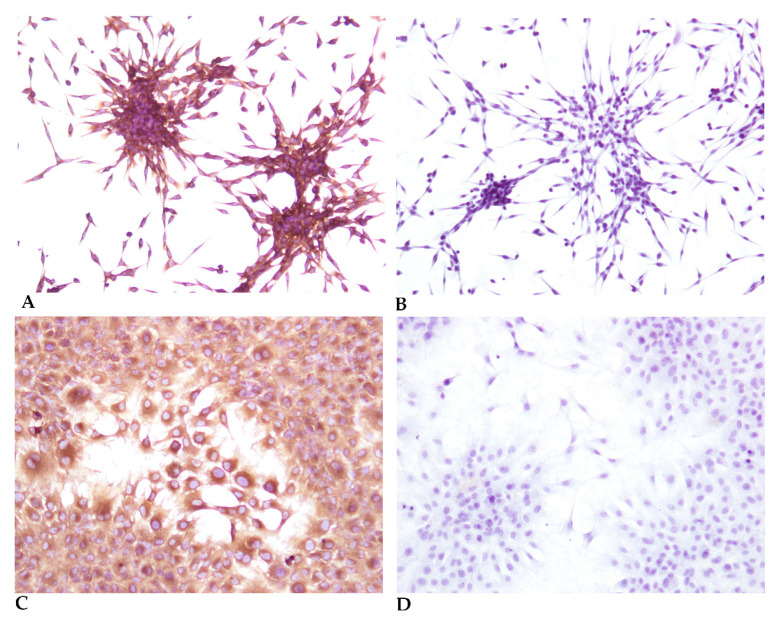
Immunocytochemistry of the third passage (subculture) (**A**) Immunocytochemistry using an anti-Vimentin Clone V9 antibody, of a 20% confluent culture, showing typical spindle cell morphology, 40×; (**B**) Immunocytochemistry using an anti-Cytokeratin AE1/AE3 antibody, of a 20% confluent culture, showing typical spindle cell morphology, 40×. Cells were fixated at lower confluence to allow a better observation of the cell morphology. (**C**) Immunocytochemistry using an anti-Vimentin Clone V9 antibody, of a 70% confluent culture, 100× (**D**) Immunocytochemistry using an anti-Cytokeratin AE1/AE3 antibody, of a 70% confluent culture, 100×.

In order to check the purity of the culture obtained and confirm the evidence given by the morphological data, immunocytochemistry was performed (Figure 4) using cells grown in 8-well slides (Thermo Scientific™ Nunc™ Lab-Tek™ Chamber Slide System). The first, second and third passages were cultivated and evaluated with 8 replicates for each species (rabbit and hare). Two primary antibodies were used, a monoclonal antibody anti-Vimentin, Clone V9 (Figure 4A,C) that marks fibroblasts, endothelial cells, lymphoid tissue and melanocytes and a monoclonal antibody anti-Cytokeratin AE1/AE3 as epithelial cells marker (Figure 4B,D).

(1) Grow cells in chamber slide system (e.g., (Thermo Scientific™ Nunc™ Lab-Tek™ Chamber Slide System)) until 95% cell confluence is reached.
(2) Remove the medium and wash 2 times with PBS.
(3) Fix cells by adding 100 μL of 100% acetone (we used Acetone, for GC residue analysis (Scharlab, Barcelona, Spain)) to each well and incubate for 10 min. Remove the acetone.
(4) Remove the slides from the chamber slide system. Rehydrate the cells in Dako EnVision FLEX Wash Buffer (Agilent, Santa Clara, CA, USA) with 1% of Triton X-100 for 5 min.
(5) Wash the cells with Dako EnVision FLEX Wash Buffer.
(6) Delimitate the cell wells with a Dako Pen.
(7) Incubate the cells with EnVision FLEX Peroxidase-Blocking Reagent for 10 min.
(8) Wash the cells with Dako EnVision FLEX Wash Buffer.
(9) Incubate the cells at RT with Monoclonal Mouse Anti-Human Cytokeratin Clones AE1/AE3 (Dako, M3515) and Monoclonal Mouse Anti-Vimentin Clone V9 (Dako, M0725), both diluted 1:100 in EnVision FLEX Antibody Diluent.
(10) Wash the slides with Dako EnVision FLEX Wash Buffer, 2 times for 5 min, with manual shaking. Change the buffer between washes.
(11) Incubate with Envision FLEX/HRP for 30 min (Dako, SM802) at RT.
(12) Wash the slides with Dako EnVision FLEX Wash Buffer, 2 times for 5 min, with manual shaking. Change the buffer between washes.
(13) Incubate with Envision FLEX DAB and Chromogen dilution in the respective buffer, for 3–5 min at RT.
(14) Wash in distilled water.
(15) Nuclei counterstaining with hematoxylin solution for 5 min at RT.
(16) Dehydrate the cells by incubation of ethanol in the following sequence: 70, 80%, 95, and 100%, 1 min each, followed by 2 baths in xylene, 1 min each.
(17) Mount the slides with Slide Mounting Media.

## 3. Discussion and Conclusions

Iberian hare and European wild rabbit are the natural hosts and reservoirs of many known multiple species-specific (e.g., myxoma virus, Shope fibroma virus) and zoonotic (e.g., Hepatitis E virus, *Francisella tularensis*) pathogens, and of others yet unidentified that need to be characterized. In this manuscript we describe, for the very first time, a simple protocol for establishing an in vitro model system of fibroblastic cells already differentiated, derived from biopsies. Moreover, this methodology can be successfully applied to tissue samples derived from healthy adult animals, or even shortly after death (up to two hours).

The isolation of fibroblasts from skin biopsies is a fairly common procedure in humans with a few protocols already available in the literature [6,8,10,11]. However, this approach is new in the field of Veterinary Medicine, since reports of virus isolation or vaccine production, including Influenza [12], Newcastle [13], Rabies [14], Pseudorabies [15], among others, used primary cultures of fibroblasts or epithelial cells, derived from tissue samples obtained during embryogenesis, or at the start of the postnatal period.

The main novelty of the in vitro model system of fibroblastic cells described here is its establishment in a short period and, most of all, the great efficiency regarding cell density, viability, and purity. The trypan blue exclusion test showed a mean cell viability of 95.2 ± 1.1% during the first four subcultures, decreasing afterwards to 73.4 ± 7.1% after the seventh subculture. No more than eight subcultures were analyzed for this purpose. The cell proliferation rate using 10 replicates was estimated at 31.8 h during the first four passages. The purity of these cell cultures was also assessed by phenotype specific cell markers, namely vimentin (Figure 4) although morphologically the cells are easily identifiable (especially with low confluence). The absence of labelling by pancytokeratin (Figure 4), excludes the presence of contaminant epithelial cells. In the first passage, a fibroblast purity of 85.3 ± 9.5% was obtained. In the second and third passages, the purity was 99.3 ± 0.6%, considering eight replicates of each passage for each species. Contamination of the fibroblast cell cultures occurred mainly by keratinocytes and other epithelial cells, probably from hair follicles and other skin adnexal structures.

The viability and cell proliferation rate of fibroblasts were similar to the values reported by other authors [10,16]. In addition, differentiated fibroblastic cells may be subcultured over time with no loss of the morphology or organization of the cell layer. Although there are some primary fibroblast cell lines from domestic rabbits commercially available, the genetic differences between both rabbit species of origin and the rabbit species understudied, and between the wild rabbit and the Iberian hare, compelled us to produce primary fibroblast cell cultures. Fibroblasts are undoubtedly the choice that best allows for the primary isolation of viruses in a homologous system and the increase in viral loads through successive replicative cycles, before adaptation to a permissive cell line, which is easier to manipulate.

In conclusion, the method described in this manuscript is a fast and cheap method to establish primary Leporidae skin fibroblast cultures, providing an alternative technique to obtain highly pure primary fibroblast cultures from rabbit and hare skin explants. This method can also be adapted for the epidermis and can be modified to obtain a pure culture of keratinocytes derived from the epidermis (results not shown).

## Figures and Tables

**Table 1:** Available techniques for primary fibroblasts isolation.

Species Used	Tissue Type	Harvest	Technique	Notes	Reference
Human	Skin biopsies from anterior surface of the forearm	HAM-F10 cell culture medium with 20% FBS	Adhesion of fragments to the flask surface	Large maintenance costs of culture medium during initial fibroblast growth.	[5]
Human	Skin biopsy	DMEM cell culture medium with 20% FBS	Adhesion of fragments to the well bottom	Estimation of 25–35 days to second passage. Large maintenance costs of culture medium during initial fibroblast growth	[6]
Human	Skin biopsy	Complete DMEM or complete RPMI	Primary explant method without/with epidermis removal with 0.5% dispase/PBS or 0.3% trypsin/PBS	Confluence is generally reached in ∼3 to 5 weeks.	[7]
Mouse	Skin from tail and ear	RPMI: 10% fetal calf serum (FCS), 50 μM 2-mercaptoethanol, 100 μM asparagine, 2 mM glutamine, 1% penicillin-streptomycin solution.	Digestion with collagenase and D-pronase solution	Euthanasia was used. The fragments correspond to epidermis and dermis to ensure the grow of keratinocytes is probable.	[8]

## Data Availability

Data are contained within the article.
